# Macrophage Migration Inhibitory Factor in *Psoroptes ovis*: Molecular Characterization and Potential Role in Eosinophil Accumulation of Skin in Rabbit and Its Implication in the Host–Parasite Interaction

**DOI:** 10.3390/ijms24065985

**Published:** 2023-03-22

**Authors:** Xiaobin Gu, You Ge, Ya Wang, Cuirui Huang, Guangyou Yang, Yue Xie, Jing Xu, Ran He, Zhijun Zhong, Deying Yang, Zhi He, Xuerong Peng

**Affiliations:** 1 Department of Parasitology, College of Veterinary Medicine, Sichuan Agricultural University, Chengdu 611130, China; 2 Institute of Animal Genetics and Breeding, College of Animal Science and Technology, Sichuan Agricultural University, Chengdu 611130, China; 3 Department of Chemistry, College of Life and Basic Science, Sichuan Agricultural University, Ya’an 625014, China

**Keywords:** *Psoroptes ovis*, MIF, sequence analysis, transcriptional level, tissue localization, skin eosinophil recruitment

## Abstract

*Psoroptes ovis,* a common surface-living mite of domestic and wild animals worldwide, results in huge economic losses and serious welfare issues in the animal industry. *P. ovis* infestation rapidly causes massive eosinophil infiltration in skin lesions, and increasing research revealed that eosinophils might play an important role in the pathogenesis of *P. ovis* infestation. Intradermal injection of *P. ovis* antigen invoked massive eosinophil infiltration, suggesting that this mite should contain some relative molecules involved in eosinophil accumulation in the skin. However, these active molecules have not yet been identified. Herein, we identified macrophage migration inhibitor factor (MIF) in *P. ovis* (*Pso*MIF) using bioinformatics and molecular biology methods. Sequence analyses revealed that *Pso*MIF appeared with high similarity to the topology of monomer and trimer formation with host MIF (RMSD = 0.28 angstroms and 2.826 angstroms, respectively) but with differences in tautomerase and thiol-protein oxidoreductase active sites. Reverse transcription PCR analysis (qRT-PCR) results showed that *Pso*MIF was expressed throughout all the developmental stages of *P. ovis*, particularly with the highest expression in female mites. Immunolocalization revealed that MIF protein located in the ovary and oviduct of female mites and also localized throughout the stratum spinosum, stratum granulosum, and even basal layers of the epidermis in skin lesions caused by *P. ovis*. r*Pso*MIF significantly upregulated eosinophil-related gene expression both in vitro (PBMC: CCL5, CCL11; HaCaT: IL-3, IL-4, IL-5, CCL5, CCL11) and in vivo (rabbit: IL-5, CCL5, CCL11, P-selectin, ICAM-1). Moreover, r*Pso*MIF could induce cutaneous eosinophil accumulation in a rabbit model and increased the vascular permeability in a mouse model. Our findings indicated that *Pso*MIF served as one of the key molecules contributing to skin eosinophil accumulation in *P. ovis* infection of rabbits.

## 1. Introduction

*Psoroptes ovis* is a common ectoparasite of both domestic and wild animals worldwide and the causative agent of psoroptic mange, which causes an inflammatory skin disease correlated with significant morbidity and significant adverse effects on the economics of the animal industry [1]. This surface-living mite abrades host skin epidermis and deposits excretory–secretory proteins onto the host stratum corneum, resulting in skin inflammation [2,3]. The histopathology of skin lesions revealed that eosinophils were the predominant cellular component of the infiltrate [4,5]. This influx of eosinophils in skin lesions correlated with disease severity evoked by *P. ovis* infestation. The number of eosinophils in skin lesions of sheep was associated with the mite population and skin lesion area [4]. These similar findings have been observed in cattles and rabbits with *P. ovis* infestations. In the susceptible breed of Belgian blue cattle, eosinophil numbers in skin lesions were more pronounced in comparison with the non-susceptible breed of Holstein–Friesian cattle [6]. In rabbits with *P. ovis* infestations, eosinophil levels showed a significant elevation in severely infected cases [7]. Moreover, the infiltration of eosinophils in skin lesions might supply sufficient food for the *P. ovis* mite to drive its population growth [8]. These studies suggested that eosinophils in skin might play an important role in the pathogenesis of *P. ovis* infestations. In addition, the similar phenomenon of massive accumulation of eosinophils in the skin was evoked by intradermal injection of *P. ovis* antigen [9]. Interestingly, *P. ovis* mite antigens were able to promote eosinophil migration in vitro [10]. Therefore, this mite should contain some molecules involved in eosinophil accumulation of the skin lesions. Unfortunately, these active molecules have not yet been identified. 

Macrophage migration inhibitory factor (MIF) has been found across a wide range of different organisms, including parasites, bacteria, plants, fish, birds, rabbits, sheep, cattle, humans, etc. [11], and it was firstly reported as a lymphokine and able to inhibit random migration of macrophages [12]. Increasing research on MIF revealed its unusual properties as distinguished from other cytokines. Parasite-derived MIF can promote eosinophil infiltration [13,14] and indirectly or directly affect the production and survival of eosinophils [15,16], suggesting that MIF plays an important role in eosinophil biology and eosinophil-mediated inflammation [17]. Recently, He et al. found the existence of MIF in *P. ovis* transcriptomic data [1], and a further report states that the recombinant *Pso*MIF protein (r*Pso*MIF) shifted the balance both Th1/Th2 and Th17/Treg in peripheral blood mononuclear cells (PBMC) [18]. Beyond that, no studies have been conducted on *Pso*MIF. 

Considering the close association between MIF and eosinophils, we speculated that *Pso*MIF may play an important role in eosinophil accumulation in skin lesions caused by *P. ovis* infection. Therefore, the aim of this study is (1) to conduct sequence analysis of orthologous MIF genes from *P. ovis* and other organisms (mites and hosts); (2) to analyze the transcriptional expression of *Pso*MIF gene and its tissue localization in *P. ovis* mites; (3) to investigate the potential roles of r*Pso*MIF in skin eosinophil accumulation in a rabbit model and evaluate the vascular permeability effects of r*Pso*MIF in a mouse model; (4) to discuss our results obtained and explore the potential implications of *Pso*MIF in the host–parasite interaction. 

## 2. Results

### 2.1. Sequence Comparison of Orthologous MIF from P. ovis and Other Organisms

**Figure 1:**
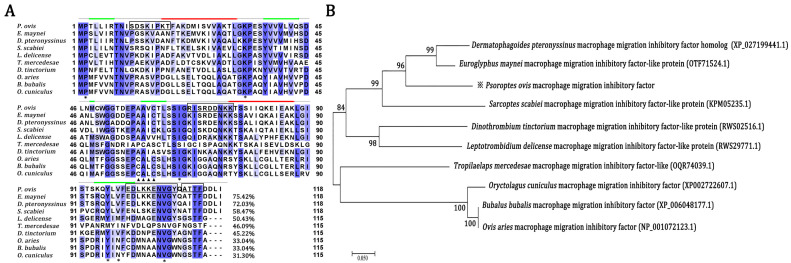
Alignment comparison of multiple sequences (**A**) and neighbor-joining (NJ) tree were constructed based on MIF amino acid sequences (**B**). (**A**) The deduced amino acid sequences of MIF were compared with homologous sequences of related proteins in other mites and hosts, including: *Euroglyphus maynei* (GenBank: OTF71524.1), *Dermatophagoides pteronyssinus* (GenBank: XP_027199441.1), *Sarcoptes scabiei* (GenBank: KPM05235.1), *Leptotrombidium delicense* (GenBank: RWS29771.1), Dinothrombium tinctorium (GenBank: RWS02516.1) and *Tropilaelaps mercedesae* (GenBank: OQR74039.1), Ovis aries (sheep) (GenBank: XP_006048177.1), *Bubalus bubalis* (Water buffalo) (GenBank: XP_006048177.1), *Oryctolagus cuniculus* (rabbit) (GenBank: XP002722607.1). Helices are marked as red tubes and sheets as dark green arrows on the sequence. The six amino acid residues in mammalian MIFs previously predicted to interact with the tautomerase substrate are marked by asterisks (*); the motif of CXXC present in mammalian MIF is marked by filling triangles (▲). Identical amino acid residues between *Pso*MIF and other MIFs are shaded blue. (**B**) Number beyond every branch is the bootstrap value of 1000 replications (%).

**Figure 2:**
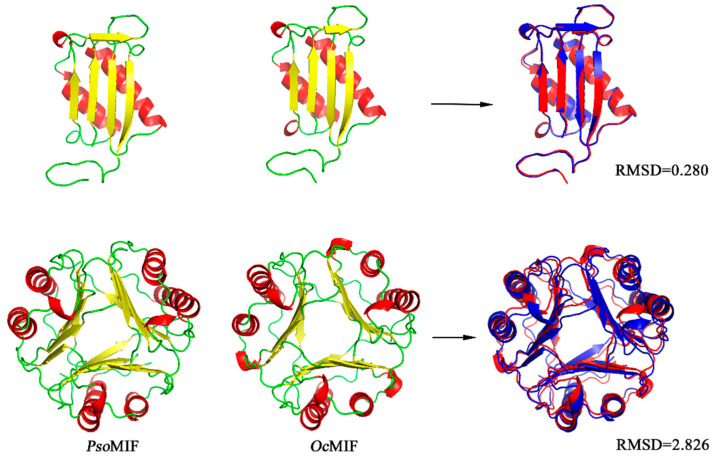
Level of structural similarity between *Pso*MIF and host MIF. Schematic representation of *Pso*MIF and *Oryctolagus cuniculus* MIF (*Oc*MIF) monomers (**top**) and trimers (**bottom**) with secondary structure elements shown in yellow (β-sheet), red (α-helix), and green (random coil). The right side shows the superposition of the backbone of monomeric and trimeric *Pso*MIF (blue) and *Oc*MIF (red).

The amino acid sequence of PsoMIF shared 45.22–75.42% identity with MIF from six other mites (Euroglyphus maynei, Dermatophagoides pteronyssinus, Sarcoptes scabiei, Leptotrombidium delicense, Dinothrombium tinctorium and Tropilaelaps mercedesae) but only shared 31.30–33.04% identity with host MIFs (sheep, cattle, and rabbit) (Figure 1A). Analyzing the amino acids in active sites, we found that PsoMIF possessed five out of six conserved catalytic-sites for tautomerase activity in the mammalian MIFs (Pro2, Lys33, Ile65, Tyr96, Val107), and Val98 in PsoMIF was substituted for Glu98 in mammalian MIFs (Figure 1A, Table 1). Similar to the other six mite MIF homologues, PsoMIF lacked the CXXC motif (Cys57-Ala-Leu-Cys60) for only a single cysteine residue (Cys60) identical to the conserved thiol-protein oxidoreductase site in mammalian MIFs (Figure 1A, Table 1). An NJ tree showed that P. ovis presented closer (Bf = 96%) to Euroglyphus maynei and Dermatophagoides pteronyssinus than to the hosts (sheep, cattle, rabbit) (Bf = 100%) (Figure 1B). Secondary and tertiary structure analysis revealed that PsoMIF comprised two α-helices and five β sheets (Figure 1A and Figure 2). Interestingly, despite very low amino acid sequence identity between PsoMIF and host MIFs (31.30–33.04%), they have significant similarity in the topology of monomer and trimer formation (RMSD = 0.28 angstroms and 2.826 angstroms, respectively; see Figure 2). 

### 2.2. Western Blotting Analysis of rPsoMIF

**Figure 3:**
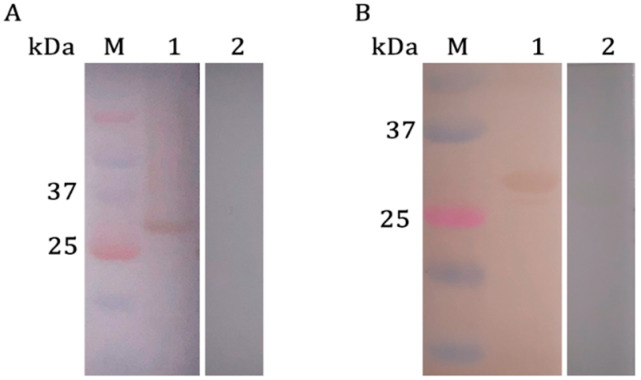
Western blotting of r*Pso*MIF protein. (**A**) Lane M: protein molecular weight marker. The purified r*Pso*MIF probed with serum from a rabbit naturally infected with *P. ovis* (Lane 1) and *P. ovis*-negative serum (Lane 2). (**B**) Lane M: protein molecular weight marker. The purified r*Pso*MIF probed with the rat anti-r*Pso*MIF-IgG (Lane 1) and negative rat serum before immunization (Lane 2).

Western blotting analysis in Figure 3 showed that r*Pso*MIF was specifically recognized by *P. ovis*-positive rabbit serum and the correspondent rat anti-*Pso*MIF IgG. However, *P. ovis*-negative rabbit serum and pre-immune rat serum could not be bound, which demonstrated the favorable reactivity of r*Pso*MIF. 

### 2.3. PsoMIF mRNA Expression Patterns in P. ovis Mites

**Figure 4:**
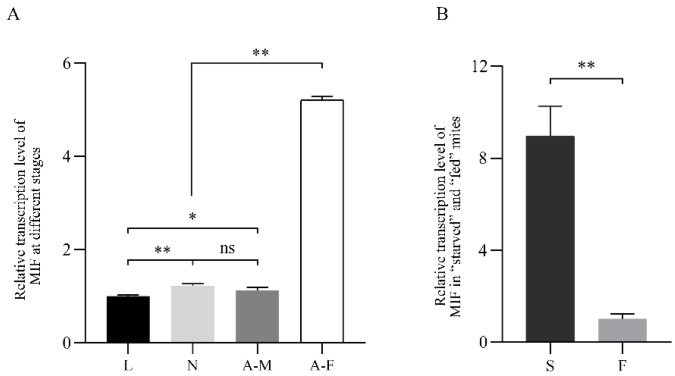
Relative transcription level of *Pso*MIF in different stages (**A**) and “starved’’ or ‘‘fed’’ mites (**B**). The internal reference gene used in this experiment is β-actin. L—larva, N: nymph, AM—adult male, AF—adult female, S—starved mites, F—fed mites. Data are expressed as mean ± SE. ns = *p* > 0.05, * = *p* < 0.05, ** = *p* < 0.01.

Figure 4 showed the *Pso*MIF transcription expression in all lifecycle stages mites and for “starved” as well as “fed” mites. Results revealed that *Pso*MIF mRNA was transcribed throughout the lifecycle of *P. ovis*, and female mites had the highest transcription level compared with male (*p* < 0.01), nymph (*p* < 0.01), and larval mites (*p* < 0.01). In addition, nymph and male mites showed a significantly higher transcription level than larval mites (*p* < 0.05, Figure 4A). However, there was no significant difference between nymph and male mites in *Pso*MIF expression (*p* > 0.05, Figure 4A). Additionally, the expression of *Pso*MIF in “starved” mites was 8.9 times higher than that in “fed” mites (*p* < 0.01, Figure 4B). 

### 2.4. Detection of Native PsoMIF in Adult Female Mites and P. ovis Skin Lesions

**Figure 5:**
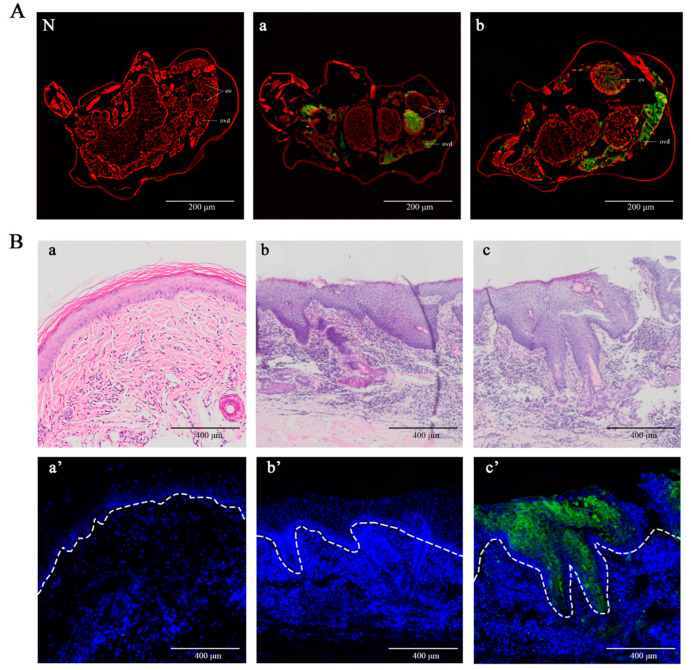
Immunolocalization of native *Pso*MIF in the adult female of *P. ovis* (**A**) and *P. ovis* skin lesions (**B**). (**A**) (N): incubate with the pre-immune IgG negative control; (a,b): incubate with the specific anti-r*Pso*MIF IgG. ov—ovary; ovd—oviduct. Scale bar = 200 μm. (**B**) Non lesions (a) and lesions of (b,c) skin sections of *P. ovis* infected rabbit stained by hematoxylin and eosin (HE); skin sections incubated with the specific anti-r*Pso*MIF-IgG (a’,c’) and pre-immune rat IgG (b’). Scale bar = 400 μm.

Immunofluorescence histochemistry was performed to detect the location of native *Pso*MIF using rat anti-r*Pso*MIF serum. Native *Pso*MIF was predominantly localized in the female mite ovary and oviduct with strong green fluorescence (Figure 5A(a,b)). In *P. ovis* skin lesions, native *Pso*MIF was mainly distributed in the stratum spinosum and stratum granulosum of the epidermis and even entered the basal layer in severe lesions (Figure 5B(c’)), while no staining was observed in non-lesional skin (Figure 5B(a’)). The pre-immune IgG showed no green fluorescence in female mites (Figure 5A(N)) and skin lesions (Figure 5B(b’)). 

### 2.5. rPsoMIF Upregulates Eosinophil-Related Gene Expression in Allergic Rabbit PBMC

The appropriate concentration of r*Pso*MIF was screened by CCK-8, revealing that r*Pso*MIF did not impair PBMC viability at four different concentrations (0.01, 0.05, 0.1, and 1 μg/mL), and 1 μg/mL r*Pso*MIF significantly promoted PBMC proliferation in vitro (*p* < 0.05, Figure S1). Thus, we chose 1 μg/mL r*Pso*MIF as the appropriate concentration for subsequent tests. 

**Figure 6:**
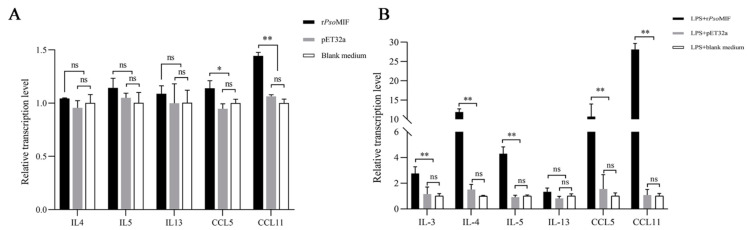
Transcription level of eosinophil-related genes in allergic rabbit PBMC (**A**) and HaCaT cells (**B**). Relative mRNA levels were normalized to β-actin for PBMC analysis as well as YWHAZ gene for HaCaT analysis. Data are expressed as mean ± SE. ns = *p* > 0.05*,* * = *p* < 0.05, ** = *p* < 0.01.

To determine whether r*Pso*MIF had impacts on the eosinophil-related gene expression in allergic rabbit PBMC, qRT-PCR analysis was performed on cells. Results revealed that r*Pso*MIF significantly upregulated the relative expressions of chemokine CCL5 (RANTES) and CCL11 (eotaxin-1) compared with pET32a and blank medium control groups (*p* < 0.05 for CCL5; *p* < 0.001 for CCL11); however, r*Pso*MIF had only slight and insignificant upregulation of cytokines IL-4, IL-5, and IL-13 compared to those in control groups (*p* > 0.05) (Figure 6A). 

### 2.6. rPsoMIF Significantly Upregulates Eosinophil-Related Gene Expression in HaCaT Cells

A CCK-8 assay was carried out to determine the appropriate concentration of r*Pso*MIF in HaCaT cells. Four different concentrations of r*Pso*MIF (0.05, 0.1, 0.5, and 1 μg/mL) processed for 24, 48, and 72 h, respectively, all promoted cell proliferative rates. There was a significant proliferation rate in 0.1, 0.5, 1 μg/mL r*Pso*MIF (*p* < 0.001, Figure S2A), and the highest proliferation rate occurred after 24 h. Therefore, 0.1 μg/mL r*Pso*MIF and 24 h were chosen in subsequent tests.

qRT-PCR analysis was used to evaluate whether r*Pso*MIF had impacts on the eosinophil-related gene expression in human immortalized-keratinocyte-line HaCaT cells. Compared to pET32a and blank medium control groups, r*Pso*MIF showed a significantly upregulated the relative expression level of chemokine CCL11, CCL5, and cytokines IL-3, IL-4, as well as IL-5 (*p* < 0.05 for IL-3; *p* < 0.001 for other target genes, Figure 6B). Among them, CCL11 showed the highest-level expression in r*Pso*MIF treatment group, and its expression was 27 times higher than in the blank medium control group. However, cytokine IL-13 showed no significant difference between r*Pso*MIF treatment and pET32a as well as the blank medium control treatments (*p* > 0.05, Figure 6B). 

### 2.7. rPsoMIF Induces Skin Inflammation and Eosinophil Recruitment In Vivo

Given the finding in in vitro experiments that r*Pso*MIF can upregulate eosinophil-related gene expression in PBMC and HaCaT cells, we further investigated whether the r*Pso*MIF is also important in skin eosinophil recruitment in rabbits.

**Figure 7:**
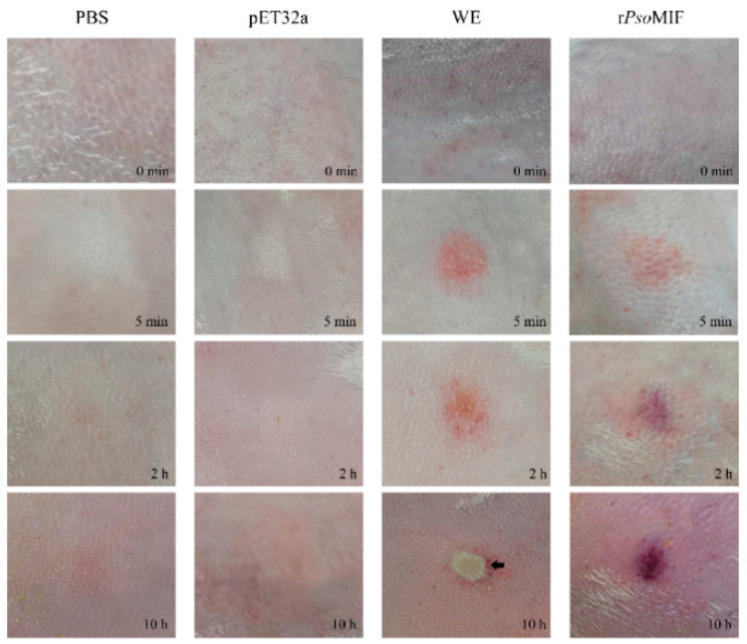
Clinical observation. Representative pictures of intradermal injection of PBS, pET32a, WE, and r*Pso*MIF in rabbits. WE—whole extract of *P. ovis*, black arrow—scab.

**Figure 8:**
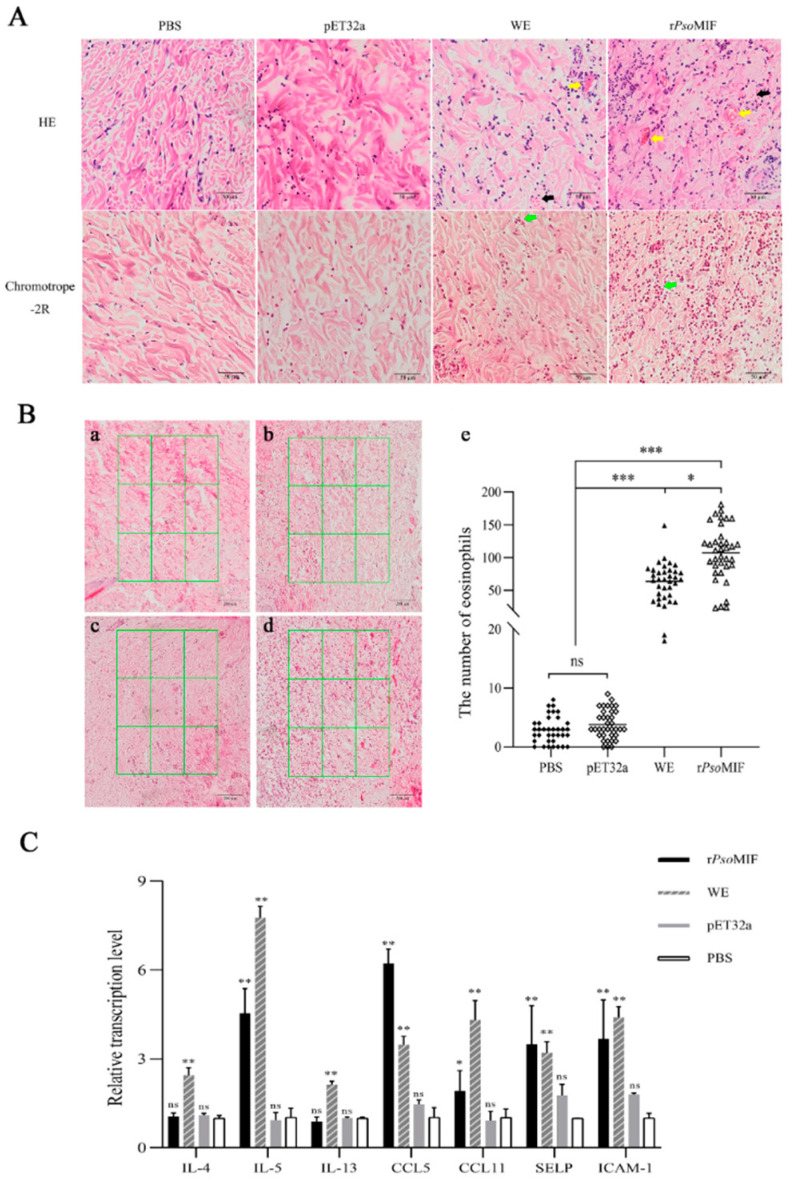
r*Pso*MIF induces skin inflammation and eosinophil recruitment in vivo. (**A**) Pathological section staining of skin. Representative pictures of hematoxylin and eosin (HE) and Chromotrope-2R stained sections of skin after intradermal injection of PBS, pET32a, WE, and r*Pso*MIF. WE—whole extract of *P. ovis*, black arrow—erythrocyte, yellow arrow—telangiectasia, green arrow—eosinophils. Scale bar = 50 μm. (**B**) Eosinophils counts in the selected area. Representative pictures of Chromotrope-2R staining of skin sections after intradermal injection with PBS (a), pET32a (b), whole extract (WE) of *P. ovis* (c), and r*Pso*MIF (d). Scale bar = 200 μm. (e)—eosinophil count. ns = *p* > 0.05, * = *p* < 0.05, *** = *p* < 0.0001. (**C**)Transcription level of eosinophilia-related genes in vivo from rabbit skin. WE—whole extract of *P. ovis*, SELP—P-selectin. ns = *p* > 0.05, * = *p* < 0.05, ** = *p* < 0.001.

We found that both r*Pso*MIF and *P. ovis* WE (whole extract) caused skin inflammation at the injection site, including obvious redness and swelling in a time-dependent manner, while PBS and pET32a treatments did not cause obvious skin reactions (Figure 7). Interestingly, *P. ovis* WE protein even became a scab at the injection site at 10 h after treatment. Similar effects were observed in histologic examination with obvious dermal telangiectasia and bleeding in r*Pso*MIF and WE treatments, while no obvious pathological changes in PBS and pET32a treatments (Figure 8A) were observed. Next, we found increased recruitment of eosinophil to the skin in chromotrope-2R staining after r*Pso*MIF and WE treatments compared with PBS and pET32a treatments, and there was statistical significance (Figure 8B). Surprisingly, the number of eosinophils at the injection skin site was significantly higher in r*Pso*MIF treatment than in WE treatment (*p* < 0.05, Figure 8B). Next, we investigated the expression of mRNAs for eosinophil-related genes in injection skin specimens from r*Pso*MIF, WE, PBS, and pET32a treatments. The expression levels of cytokine IL-5, chemokines CCL5, CCL11, and adhesion receptors P-selectin and ICAM-1 were significantly increased in r*Pso*MIF treatment compared to PBS and pET32a treatments (*p* < 0.05; *p* < 0.001, Figure 8C). In contrast, no significant difference in cytokines IL-4 and IL-13 between r*Pso*MIF treatment and PBS as well as pET32a treatments were detected (*p* > 0.05, Figure 8C). WE treatment had a statistically significant higher-level expression in all target genes in comparison with PBS and pET32a treatments (*p* < 0.001, Figure 8C). 

### 2.8. rPsoMIF Enhances Vascular Permeability

**Figure 9:**
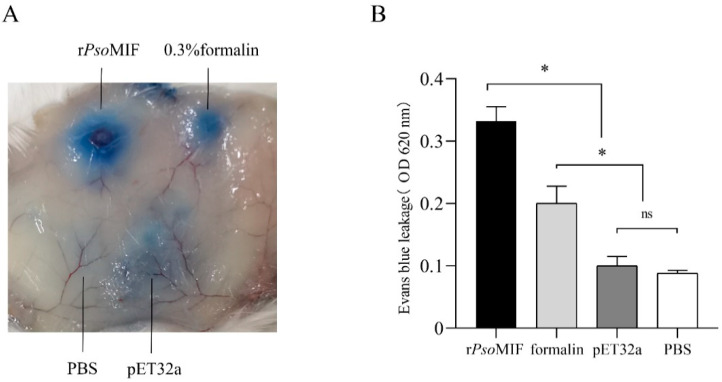
Effects of r*Pso*MIF on vascular permeability. Mice were injected intradermally with 20 μg r*Pso*MIF, 20 μg pET32a, 0.3% formalin. PBS was used as a vehicle control (*n* = 6). (**A**)—the representative photo of Evans blue leakage into the injection site. (**B**)—Evans blue extravasation was estimated after extraction with formamide and reading at absorbance 620 nm. ns *p* > 0.05, * *p* < 0.05.

Additionally, we evaluated whether r*Pso*MIF could affect the vascular permeability using the Miles assay by observing vascular leakage of Evans Blue dye in mouse skin. Figure 9 showed that r*Pso*MIF significantly increased the vascular permeability into skin subcutaneous tissue in comparison with 0.3% formalin, pET32a, and PBS (*p* < 0.05).

## 3. Discussion

Despite the increasing evidence that mammalian MIF contributes to the pathology in many inflammatory diseases [10], the role of *P. ovis* homolog of MIF in psoroptic mange pathology is poorly described. Here, we conducted a sequence comparison of *Pso*MIF and other organism MIFs and evaluated the transcriptional profile of *Pso*MIF and its tissue localization in *Psoroptes* mites and infected rabbit lesional skin. Particularly, we investigated the potential roles of r*Pso*MIF in skin eosinophil accumulation both in vitro and in vivo. Our results showed that *Pso*MIF appeared a high similarity in the topology of monomer and trimer formation with host MIF but with differences in tautomerase and hiol-protein oxidoreductase active sites. *Pso*MIF was transcribed by all developmental stages, and its native protein was mainly localized in the ovary and oviduct of female mites as well as in the epidermis of lesional rabbit skin. Furthermore, r*Pso*MIF enhanced the vascular permeability and significantly induced eosinophil accumulation in the skin. To our knowledge, this study is the first report of antigen molecules of *Psoroptes* mite inducing eosinophil accumulation in the skin lesions of psoroptic mange. 

Despite limited homology, *Pso*MIF exhibited the same secondary structure distribution of two α-helices and five β-strand sequences (β1α1β2β3β4α2β5) with host MIF, and it appeared a similar topology of monomer and trimer formation with host MIF (Figure 1A). However, there were differences in catalytic active sites between *Pso*MIF and host MIFs. *Pso*MIF possessed five out of six amino acid residues known as the conserved active site for MIF’s tautomerase activity [19], suggesting that *Pso*MIF was accessible for interaction with tautomerase substrates. This speculation was supported by the result of enzyme activity assays in our previous study, revealing that r*Pso*MIF possessed tautomerase activity [17]. However, single-amino-acid mutations in active sites might significantly affect MIF enzymatic activity [20]. *Pso*MIF appeared a single-amino-acid difference at position 98 from mammalian MIFs, and this change could be related to lower levels of tautomerase activity in *Pso*MIF than that of human MIF [18] and might also account for mammalian MIF-specific antagonist Z950 only having a slight inhibitory effect on *Pso*MIF activity [18]. This dissimilarity in tautomerase active sites might allow for the development of specific inhibitors for parasite rather than host MIF, and this inhibitor targeting *Pso*MIF might act as a novel agent for therapeutic intervention [21]. Additionally, three out of four amino acid residues (CXXC motif) for MIF’s thiol-protein oxidoreductase activity [22] were mutated in *Pso*MIF, possibly explained the lack of oxidoreductase activity of r*Pso*MIF in vitro [18].

Previous studies documented that parasite-derived MIF, including *Plasmodium*, *Toxoplasma,* and *Ostertagia ostertagi*, had an important role in their life cycle stage development [23,24,25]. Our transcriptional analysis data in this study supported this opinion. In the present study, we found that *Pso*MIF was expressed in all developmental stages of *P. ovis*. Interestingly, both gene transcript and native protein of *Pso*MIF were in high abundance at the female stage mites, particularly this native protein was localized to the ovary and oviduct of female mite, indicating that this MIF might be associated with female mite reproduction physiology. Additionally, MIF has been reported to enhance *Myzus percicae* and *Acyrthosiphon pisum* feeding [26], so we suggested that the high abundance expression of MIF in female mites might be related to this stage need for feeding a large amount of food to acquire nutrients before oviposition [26]. Additionally, MIF appeared with a higher transcript expression in starved mites, suggesting that *Pso*MIF may be beneficial for enhancing *P. ovis* survival under malnourished conditions [23,27] or seeking host or initiating feeding behavior [28], and that this similar function of MIF could increase parasite survival under adverse nutrient stress condition has been confirmed in *Trichomonas vaginalis* [27]. Several studies reported that MIF was clearly detectable in excretory/secretory products of the parasite [25,29], and the MIF was secreted by the parasite into host tissue and blood during infection [30]. We found a very similar outcome in this study, where *Pso*MIF was released by *Psorotpes* mites during infection, and this view was supported by the following evidence in our study: (i) anti-*Pso*MIF antibodies were detected in *P. ovis*-positive serum but not in *P. ovis*-negative serum; (ii) native *Pso*MIF protein was detected throughout the stratum spinosum, stratum granulosum, and even basal layers of the epidermis in lesional skin. Interestingly, the predominant cell type in the epidermis is the keratinocyte [31], and this cell was the first contact point between *Psoroptes* mites and host immune response [32]. So, we hypothesized that *P. ovis* secreted native *Pso*MIF to contact with keratinocyte to influence the cytokine secretion and thus induce a cutaneous inflammatory response leading to the host’s skin lesions. As expected, the injection skin showed redness and swelling within 5 min after r*Pso*MIF injection (Figure 7), and these skin symptoms were similar to the early signs of *P. ovis* infection [33]. Additionally, these results suggested that *P. ovis* might secrete native *Pso*MIF to inhibit host macrophage migration into skin infection sites [13], thus allowing this mites escape detection by host’s innate immune system.

Under normal conditions, eosinophils are present almost exclusively in the gastrointestinal mucosa but not in most other healthy tissues [34]; however, under certain disease conditions, eosinophil scan selectively accumulates in any tissue of the body [34]. Eosinophils develop in the bone marrow from CD34+ pluripotent progenitor stem cells and differentiate into unique eosinophil-lineage-committed progenitors (EoPs), and then these cells develop into mature eosinophils in the bone marrow [35]. Mature eosinophils are released from the bone marrow and mobilize to the peripheral blood, then traffic rapidly from blood to tissue (including roll, adhere, and trans-endothelial migration) [36]. This multistage process of the abnormal accumulation of eosinophils in tissue is exquisitely regulated by cytokines IL-3, GM-CSF, and IL-5 [37], adhesion molecules P-selectin and intercellular adhesion molecule 1 (ICAM-1), and vascular-cell adhesion molecule 1 (VCAM-1) [38], as well as chemoattractants IL-4, IL-5, IL-13, CCL5, and CCL11 [39,40]. So, in this study, we investigated these relative molecules in vitro and in vivo. In in vitro study, the significantly increased expression of IL-5 was found in HaCaT cells but not in PBMC after exposure to r*Pso*MIF, and this cytokine is the most specific factor in all aspects of eosinophil proliferation, differentiation, migration, activation, and survival in tissues [38]. In addition to IL-5, IL-3 was also significantly upregulated in r*Pso*MIF-treatment of HaCaT cells, and this cytokine, combined with IL-5, has an additive effect on eosinophil colony production from the bone marrow [39,40]. Meanwhile, IL-4 showed a significant upregulation in r*Pso*MIF-treatment HaCaT cells, and this cytokine in cooperation with IL-5 promotes eosinophil differentiation and survival [36]. Many studies showed that the increased expression of IL-4 and IL-5 can induce the increased production of C-C chemokines (CCL5, CCL11, MCPs), and these chemokines are implicated in eosinophil trafficking from blood to tissue [39,40]. As expected, cells both in vitro (PBMC and HaCaT cells) and in vivo (rabbits) after r*Pso*MIF-treatment exhibited significant upregulation of CCL5 and CCL11. CCL5, along with IL-5, are specific selective factors for eosinophils and cooperatively promote recruitment of eosinophils into the local tissue. CCL11 involved in early recruitment of eosinophils into the tissue. Additionally, r*Pso*MIF can upregulate the adhesion receptor P-selectin and ICAM-1 in rabbits, and these molecules mediated eosinophil transendothelial migration [41]. However, it is noteworthy that P-selectin and ICAM-1 are adhesion molecules not only for eosinophil but also for neutrophils [42] and T lymphocytes [43]. In particular, r*Pso*MIF induced higher expression levels of all target genes in HaCaT cells than in PBMCs cells, which suggested that native MIF secreted by *Psoroptes* mites mainly affects the cytokines and chemokine secretions of keratinocytes, leading to the accumulation of cutaneous eosinophils at the site of infection. Interestingly, r*Pso*MIF caused a stronger accumulation of eosinophils than in WE proteins of *P. ovis*, illustrating that *Pso*MIF was one of the key proteins involved in promoting eosinophil accumulation in *P. ovis* infestation.

It is well documented that increased vascular permeability leads to edema and favors leukocyte transendothelial migration [44]. Thus, we evaluated the vascular permeability of r*Pso*MIF by the Miles assay in a mouse model. Our study revealed that r*Pso*MIF evoked increased vascular permeability leading to the serous and lymph exudations into the skin, and these exudations supply sufficient food for *P. ovis* [45]. Meanwhile, the increased vascular permeability by r*Pso*MIF potentially leads to increased eosinophil transendothelial migration from the blood vessel to skin [44], and this speculation has been confirmed in vivo in rabbits. An earlier study reported the detection eosinophils within the midgut of *P. ovis* [46], so we suggested eosinophils in the skin may serve as a food source for driving the *Psoroptes* mite population growing at the infected site. This similar beneficial role of eosinophils in the parasite survival has been characterized in *Trichinella spiralis*, revealing that eosinophils was necessary for *Trichinella spiralis* larvae growth in skeletal muscle [47]. On the other hand, eosinophils are also demonstrated to attack and kill parasites [48]. Taken together, eosinophils have a paradoxical effect on parasites, so further research is needed to elucidate the potential role of eosinophils in the pathogenesis of *Psoroptes* mite infection.

## 4. Materials and Methods

### 4.1. Sequence Comparison of Orthologous MIF from Psoroptes ovis and Other Organisms

Orthologous MIF genes from *P. ovis* and six other mites and three species mammals were extracted from GenBank and performed multiple sequence alignment by Jalview version 2.11.1 (Dundee, UK) and the online BLASTp tool (https://blast.ncbi.nlm.nih.gov/Blast.cgi, accessed on 1 June 2022). Phylogenetic tree was inferred using the neighbor-joining (NJ) method with MEGA version 7.0 through 1000 bootstrap replicates and Poisson correction method. The protein secondary and third structure of *Pso*MIF were predicted using Jpred 4.0 (http://www.compbio.dundee.ac.uk/jpred/, accessed on 20 June 2022) and SWISS Model (https://swissmodel.expasy.org/interactive, accessed on 25 June 2022), respectively. The predicted three dimensional structures were furtherly evaluated their veracity by SAVES v6.0 (https://servicesn.mbi.ucla.edu/SAVES/, accessed on 28 June 2022). Predicted tertiary structures were visualized and aligned using PyMol software. 

### 4.2. Mite Collection, Preparation of Recombinant PsoMIF (rPsoMIF), and Whole Mite Protein

A rabbit infestation with *Psoroptes ovis* var. *cuniculi* was maintained at the Department of Parasitology, Sichuan Agricultural University (Chengdu, China). The collection of *Psoroptes* mites from ear scabs was performed as described previously by Zhang et al. [49]. Mites from the different life-stage (larva, nymph, adult male, and female mites) were distinguished according to the morphological characteristics described [50]; then, the mites of each lifecycle stage and the mixed population pooled with each life-stage mite were respectively harvested. The “fed” and ‘‘starved’’ *P. ovis* mites were collected essentially according to the protocol of McNair [28]. Briefly, the mixed-stage “fed” mites were immediately harvested from the freshly ear scabs of rabbit. The freshly mites were placed in a cardboard sprayed with sterilized saline and incubated at 25 °C for 4 days under 80–90% relative humidity, then the live mites were harvested as the “starved” mites. 

Each life-cycle stage mite and “fed” and “starved” mites were used to extract total RNA using the EASYspin RNA micro kit with DNase I (Aidlab Biotechnologies Co., Ltd., Beijing, China) for the following qRT-PCR analysis. The whole mite protein (WE) was extracted from 100 mg stage-mixed *P. ovis* with BBproExtra™ total protein extraction kit (BestBio, Shanghai, China) for in vivo study on New Zealand rabbit. Additionally, the adult female mites were also involved in immunolocalization analysis. 

The purification of r*Pso*MIF was obtained as previously described by Zheng et al. [18]. r*Pso*MIF was treated using Endotoxin Removal Kit (Smart-Life Sciences Biotechnology Co., Ltd., Changzhou, China) to remove endotoxin. 

### 4.3. Polyclonal Antibody Production and Western Blotting Analysis

Anti-serum against r*Pso*MIF was raised in rats by subcutaneously injecting with 300 μg r*Pso*MIF emulsified in an equal volume of Freund’s complete adjuvant (Sigma, St. Louis, MO, USA). Following the first inoculation, three 300 μg boosters of r*Pso*MIF emulsified in Freund’s incomplete adjuvant (Sigma, USA) were applied at 7-day intervals. Serum was collected before inoculation and 4 days after the forth inoculation. Anti-r*Pso*MIF IgG was purified using Protein G affinity chromatography (Protein G Resin FF Prepacked Column, L00681, GenScript, Nanjing, China).

The purified r*Pso*MIF was subjected to 10% SDS-PAGE and eletrophoretically transferred onto a polyvinylidene difluoride (PDVF) membrane for Western blotting analysis. The membranes were blocked with 5% (*w*/*v*) skimmed milk in phosphate-buffered saline (PBS) for 2 h at 37 °C, then washed three times (5 min/wash) in TBST (0.02 M Tris-HCl, pH 7.6, 0.15 M NaCl, 0.05% Tween-20) and incubated overnight with the primary antibody (*P. ovis*-positive rabbit serum, *P. ovis*-negative rabbit serum, rat anti-r*Pso*MIF-IgG, and pre-immune serum) at 1:200 dilution in PBS at 4 °C, respectively. After washing three times in TBST, the membrane was incubated with horseradish-peroxidase (HRP)-conjugated goat anti-rabbit IgG or goat anti-rat IgG (1:3000 dilution in PBS; ABclonal Technology Co., Ltd., Wuhan, China) for 2 h at room temperature. Following four washes with TBST, the signal was detected using an Enhanced HRP-DAB Chromogenic Substrate Kit (Tiangen Biotech Co., Ltd., Beijing, China).

### 4.4. Immunolocalization of MIF in Adult Female P. ovis var. cuniculi and Lesional Skin of Rabbit Infested with P. ovis var. cuniculi

For immunolocalization analysis, a protocol was performed as previously described from Gu et al. [51]. In brief, the adult female mites were fixed in 0.8% molten. The 8 mm punch biopsy was harvested using a biopsy punch (HEAD boitechnology Co., Ltd., Beijing, China) from the advancing border of the ear lesional skin of a rabbit naturally infested by *P. ovis* var. *cuniculi* following the administration of an anesthetic (JiaBo pharmaceutical Co., Ltd., Qingyuan, China). Another 8 mm punch biopsy as the control was collected from the ear skin of a healthy rabbit. Both mites and skin biopsies were fixed in 4% polyformaldehyde, then embedded in paraffin and sliced into 5 μm sections. The sections were incubated with rat anti-r*Pso*MIF IgG or pre-immune IgG (1:200 dilution in PBS) at 4 °C overnight. After four washes with PBS, sections were incubated with fluorescein isothiocyanate (FITC)-conjugated goat anti-rat IgG (ABclonal Technology Co., Ltd., Wuhan, China; 1:2000 dilution in PBS) at 37 °C 30 min. Then, the sections were visualized by a fluorescent microscope (BX53; Olympus Corp., Tokyo, Japan). 

### 4.5. In Vitro Treatment of Rabbit PBMC

The rabbit PBMC was isolated from the blood of four allergic rabbits with the Ficoll–Hypaque centrifugation method (Solarbio Co., Ltd., Beijing, China). The allergic rabbits was performed according to previously described procedures [52]. Briefly, rabbits severely infested with *P. ovis* var. *cuniculi* were subcutaneously injected with ivermectin. Thirty days later, these treated rabbits were for clinical trials. The freshly isolated rabbit PBMC was re-suspended to a density of 1 × 10^4^ cells/mL in RPMI 1640 medium containing 1% penicillin/streptomycin solution and 10% fetal bovine serum (FBS) and seeded in a 96-well culture plate (Corning, New York, NY, USA). Cells were treated with endotoxin-free r*Pso*MIF protein (0, 0.01, 0.05, 0.1 and 1μg/mL) for 24 h at 37 °C in a 5% CO_2_ atmosphere. 10 μL/well of cell counting kit-8 (CCK-8) solution (Meilunbio, Dalian, China) were added for 1 h, and the optical density (OD) at 450 nm was detected using a microplate reader (Multiskan Spectrum, Thermo Fisher scientific, Waltham, MA, USA).

In subsequent experiments, the freshly isolated PBMC (1 × 10^6^ cells/well) was seeded in 12-well plates and treated with the optimum concentration of endotoxin-free r*Pso*MIF by CCK-8 assay. The pET32a protein or blank medium were set as control groups. Three biological replicates were applied to each group. After incubation for 24 h, cells were harvested for qRT-PCR analysis 

### 4.6. In Vitro Treatment of HaCaT

HaCaT (human immortalized keratinocytes) from Meisen CTCC (Jinhua, Zhejiang, China) were seeded in a 96-well culture plate (Corning Costar, Corning, NY, USA) at a density of 5×10^3^ cells/well, grown in a DMEM medium (Cytiva, Marlborough, MA, USA) supplemented with 10% FBS and 1% penicillin/streptomycin solution (DMEM-10) at 37 °C with 5% CO_2_ in a humidified atmosphere at sub-confluence to prevent differentiation. Treatments were carried out in DMEM supplemented with 0.5% FBS and 1% penicillin/streptomycin solution (DMEM-0.5).

For investigation the effect of r*Pso*MIF on cell viability, we plated HaCaT in 96-well plates at 5 × 10^3^ cells per well in DMEM-10 and cultured them for 1 day until reaching approximately 50% confluence, when the media was replaced with DMEM-0.5. The following day, the cells were stimulated with 5 μg/mL LPS for 24 h, and then the different concentrations of r*Pso*MIF (0, 0.05, 0.1, 0.5, and 1 μg/mL) were added. After incubation for 24 h at 37 °C in a 5% CO_2_ atmosphere, CCK-8 solution (10 μL/well) (Meilunbi) was added for 1 h to measure cell viability. The optical density (OD) at 450 nm was measured using a microplate reader (Multiskan Spectrum). 

After analysis of HaCaT viability, we seeded HaCaT into a 24-well plate at the density of 1 × 10^4^ cells/well. After stimulation with LPS (5 μg/mL) for 24 h, the optimum concentration of endotoxin-free r*Pso*MIF (0.1 μg/mL), which was screened by CCK-8 analysis, was added to each well for another 24 h. An equivalent concentration of pET32a protein or an equal volume of medium was both as the control groups. Three biological replicates were performed for each group. The cells were collected for qRT-PCR analysis. 

### 4.7. In Vivo Treatment of New Zealand Rabbits

Four allergic rabbits with a history of psoroptic mange were prepared for the present experiment as previously described [52]. The back of four allergic rabbits was shaved, and four injection locations were marked on the shaved back section. The distance between two injection sites were spaced at least 5 cm apart. Each site was injected intradermally with a volume of 0.1 mL of endotoxin-free r*Pso*MIF (1 mg/mL) or pET32a (1 mg/mL) or the whole mite extraction (1 mg/mL) or 0.01 M PBS (pH 7.0). The representative photographs of the skin injection site were recorded at four different time points (0 min, 5 min, 2 h, and 10 h). Rabbits were sacrificed at 10 h after injection; then, 8 mm-diameter skin tissue centered on the injection site was harvested for qRT-PCR and histological observation.

### 4.8. qRT-PCR

Total RNA was extracted from PBMC or HaCaT cells or rabbit skin samples by using RNA extraction kit (Foregene, Chengdu, China). qRT-PCR was performed for total RNA from each lifecycle stage mites or “fed” mites or “starved” mites or PBMC or HaCaT cells or rabbit skin samples. Primer sequences used in this study are listed in Table S1. Gene transcription was assessed by LightCycler^®^ 96 System (Roche, Basel, Switzerland) using the Real Time PCR EasyTM-SYBR Green I (Foregene, Chengdu, China). Each sample was performed in triplicate. Relative gene expression levels were examined by the 2^−ΔΔCT^ method. Relative mRNA levels were normalized to the housekeeping gene β-actin for mite, skin, and PBMC analysis [53], and the YWHAZ gene was normalized for HaCaT analysis [53]. 

### 4.9. Skin Histopathology

All skin tissues were fixed in 4% paraformaldehyde and then buried in paraffin blocks. The tissues were sliced into 5 μm sections for pathological analysis with hematoxylin-eosin staining and for eosinophils counts by Chromotrope-2R staining [54]. The stained slides were scanned with Olympus VS120 S6 Slide scanner (OLYMPUS, Tokyo, Japan). The measurements were carried out using ImagePro Plus 6.0. The number of eosinophils in scanned files was counted from 9 consecutive square areas (each at 0.09 mm^2^ area) for each sample [54].

### 4.10. Vascular Permeability Assay

Vascular permeability assay in 7-week-old BABL/c mice (*n* = 6) was performed as previously described [55]. Under anesthesia, mice were injected 100 μL Evans Blue dye (0.5% *w*/*v* in PBS) into the tail vein. After 30 min, mice were injected intradermally on the back marked sites (20 μL final volume) with (i) PBS (0.01 M, pH 7.0), (ii) formalin 0.3% in PBS, (iii) 20 μg of endotoxin-free r*Pso*MIF in PBS, or (iv) 20 μg of endotoxin-free pET32a in PBS. After 30 min, animals were euthanized and the area of skin that contained the entire injection region were removed and photographed to document leakage of the Evans Blue dye. The Evans Blue dye in the skin was extracted with 250 μL of 50% formamide overnight at 55 °C, and absorbance was measured at 620 nm after centrifugation at 4000 rpm for 10 min.

### 4.11. Statistical Analysis

All data were recorded as mean ± standard error (SE). All statistical analyses were performed with SPSS Statistics 27.0. Transcriptional analysis and eosinophil counts were evaluated by one-way analysis of variance (ANOVA) with Tukey–Kramer test. Vascular permeability was calculated by the fold difference of readings from permeability-inducing agent versus vehicle control. *p*-value < 0.05 was considered as statistically significant.

## 5. Conclusions

In conclusion, *Pso*MIF was expressed throughout all the developmental stages of *P. ovis*, particularly with the highest expression in female mites. Native *Pso*MIF was located on the ovary and oviduct of female mites and secreted into skin lesions. r*Pso*MIF significantly enhanced eosinophil-related gene expression both in vitro and in vivo, and this protein could induce cutaneous eosinophil accumulation in rabbits and increased the vascular permeability in mice. These findings suggested that *Pso*MIF served as one of the key molecules contributing to skin eosinophils accumulation during *P. ovis* infection in rabbits.

## Figures and Tables

**Table 1:** Comparison of the enzymatic active sites of *Pso*MIF with MIF from six other mites and three hosts.

ID	Tautomerase Active Site						Thiol-Protein Oxidoreductase Active Site			
Amino Acid Position	P2	P33	P65	P96	P98	P107	P57	P58	P59	P60
*O. aries* (sheep)	P	K	I	Y	N	V	C	A	L	C
*B. bubalis* (Water buffalo)	P	K	I	Y	N	V	C	A	L	C
*O. cuniculus* (rabbit)	P	K	I	Y	N	V	C	A	L	C
*P. ovis*	P	K	I	Y	V	V	A	A	V	C
*E. maynei*	P	K	I	Y	V	V	A	A	I	C
*D. pteronyssinus*	P	K	I	Y	V	V	A	A	I	C
*S. scabiei*	P	K	I	Y	V	V	A	A	I	C
*L. delicense*	P	K	I	Y	M	V	A	A	V	V
*T. mercedesae*	P	K	S	M	I	N	P	C	A	S
*D. tinctorium*	P	K	I	Y	V	V	A	A	I	A

Note: Highly conserved residues and motifs are highlighted in gray shade.

## Data Availability

The datasets supporting the conclusions of this study are included in this article.

## Supplementary Materials

The following supporting information can be downloaded at: https://www.mdpi.com/article/10.3390/ijms24065985/s1.
