# Supplementary material for: Macrophage Migration Inhibitory Factor in Psoroptes ovis: Molecular Characterization and Potential Role in Eosinophil Accumulation of Skin in Rabbit and Its Implication in the Host–Parasite Interaction
Source: Int J Mol Sci. 2023 Mar 22;24(6):5985. doi: 10.3390/ijms24065985 (PMC10059829; doi:10.3390/ijms24065985)

Supplement

Table S1 ： Primer information of genes from human and rabbit

Species	Genes (GenBank No.)	Primers	Size (bp)
**Human**	CCL5（NM_001278736.2）	F：ATTTGCCTGTTTCTGCTTGCTCTTG<br>R：AACTGCTGCTGTGTGGTAGAATCTG	106
	IL-5（NM_000879.3）	F：GAGCTGCCTACGTGTATGCCATC<br>R：ATGAGTAGAAAGCAGTGCCAAGGTC	83
	IL-4（BC067514.1）	F：ACAGCAGTTCCACAGGCACAAG<br>R：CGTACTCTGGTTGGCTTCCTTCAC	115
	IL-13（BC096140.3）	F：CAGAGGATGCTGAGCGGATT<br>R：ACTGGGCCACCTCGATTTTG	94
	IL-3（NM_000588.4）	F：CCTGCCGATCCAAACATGAG<br>R：GTCTGGGTCATGGGAGCTTG	89
	CCL11（NM_002986.3）	F：TCCCAACCACCTGCTGCTTTAAC<br>R：ACATTTGCCACTGGTGATTCTCCTG	89
	YWHAZ（NM_001135702.2）	F：ACTTTTGGTACATTGTGGCTTCAA<br>R：CCGCCAGGACAAACCAGTAT	94
**Rabbit**	CCL5 (XM_017349037.1)	F：TGCCTCGACAACAGGAAACC<br>R：ACCCCCATAGGAACCCCATA	129
	IL-5 (XM_002710201.3)	F：GTCGAAGGCAAACACTGCAC<br>R：GCTCATGCGGATTTCTGTGG	112
	IL-4 (NM_001163177.1)	F：GCGACATCATCCTACCCGAA<br>R：CTCGGTTGTGTTCTTGGGGA	116
	IL-13 (XM_002710092.3)	F：CCTTGGCAGCCTCGTATCC<br>R：GAGTGGAGCCTTCTGGTTGT	103
	CCL11 (NM_001322432.1)	F：CCCACCATCTGGCTTCCTTA<br>R：TGGGATGTGGAGAGTGGAGA	89
	SELP (selectin P)<br>(NM_001361473.1)	F：GGTAATGGCTGGAACCCTCC<br>R：AGGGATGGGGTCAAATGCAG	136
	ICAM-1 (AB128157.1)	F: TCAGGACCCTAGTCGGAAGA<br>R: ACAAGGGCTGTCACTGTTCAA	127
	β -actin (NM001101683.1)	F：GGCATGGAGTCGTGTGGCATC<br>R：CGTGTTGGCGTACAGGTCCTTG	90

Figure S1. Effects of r*Pso*MIF on the cell viability of PBMC. ns $P > 0.05$, $*P < 0.05$.

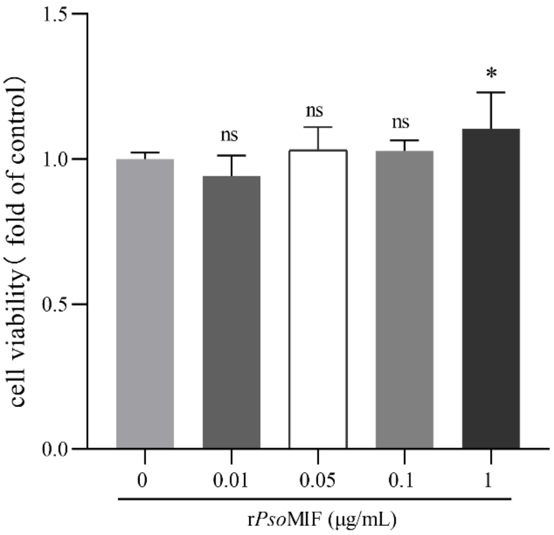

Figure S2. Effects of r*Pso*MIF on the cell viability of HaCaT. (A) Different concentrations of r*Pso*MIF; (B) Different exposure times. ns $P > 0.05$, $*P < 0.05$, $**P < 0.001$.

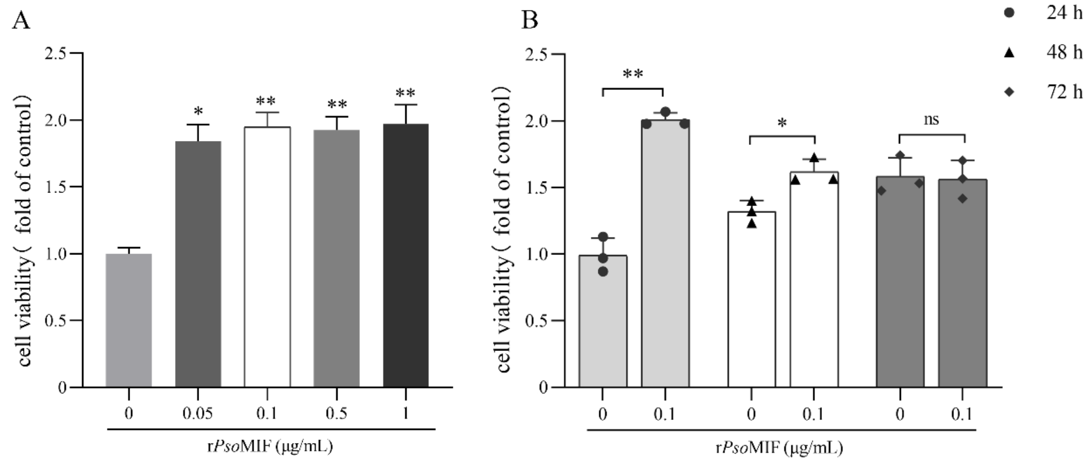

Supplement: Supplementary file 1 [file ijms-24-05985-s001.zip › ijms-2267860-supplementary.pdf]
